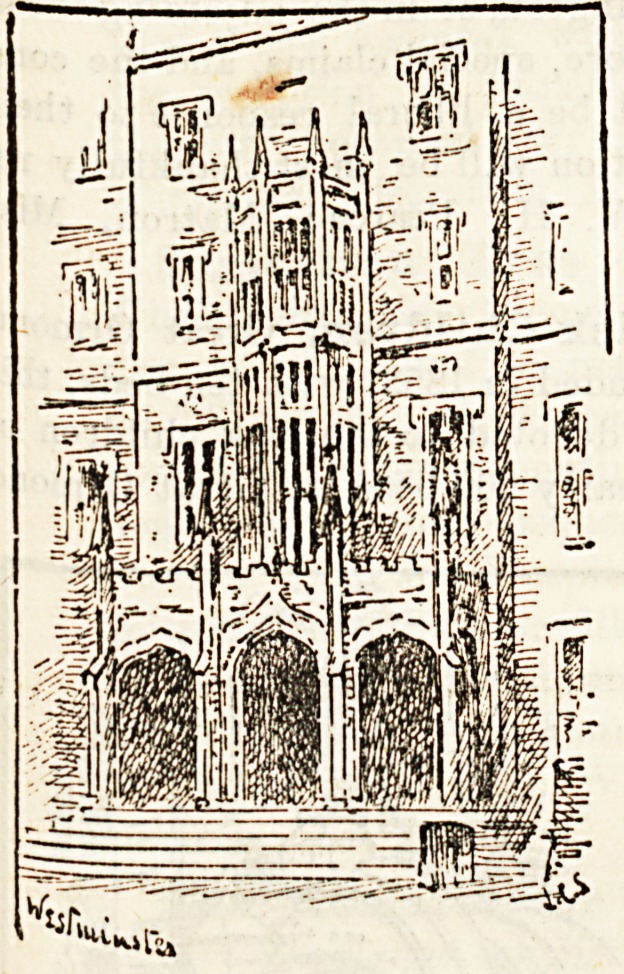# General Hospitals

**Published:** 1893-12-23

**Authors:** 


### SWEET CHARITY'S GUIDE FOR CHRISTMAS GIVERS.

### Being the Christmas Number of "The Hospital."

### GENERAL HOSPITALS.

**Charing Cross Hospital**, Agar Street, West Strand. —This institution is situated in the centre of the most crowded thoroughfares in the metropolis, and has therefore to provide for a greater number of accidents, *i.e.*, urgent cases, than probably any other hospital of its size. All such cases are immediately admitted without delay or difficulty. About 23,500 patients were relieved during the past year, more than two-thirds of which were cases of accident or emergency. Can any stronger appeal be made than this to the many visitors who usually come to London, or to the public generally? We think not. Yet, the need for pecuniary help is great and pressing, and the Council earnestly appeal for donations towards meeting the deficiency and for annual subscriptions. The present debt amounts to £9,000, and there are no investments which can be realised to relieve the hospital of this heavy burden. Secretary, Mr. A. E. Reade; Matron, Miss H. Gordon.

**German Hospital**, Dalston, N.E.—The hospital contains 120 beds, which are nearly always occupied, and admits into its wards, without any recommendation, all who are conversant with the German language (without any distinction of nationality or creed), as well as all persons who have sustained injuries from accidents. There are also a few private rooms set apart, into which patients are admitted for a weekly payment of 1½ to 2 guineas. This is a great boon to foreigners who are away from their families and cannot have proper nursing in their apartments, as well as to those who come on a visit to the metropolis and have the misfortune to fall ill, and would, but for this accommodation, have to remain in hotels at great inconvenience and expense. The grand total of the expenditure amounts to about £9,800, whilst the more reliable income of the hospital, derived from the real and funded property, annual subscriptions, Hospital Sunday and Saturday Funds, &c., amounts to about £5,600, leaving £4,200 to be collected by the hospital. Superintendent and Secretary, Mr. H. Gülich; Matron, Miss Christiane Bürger.

**Great Northern Central Hospital**, Holloway Road, N.—This hospital is entirely free to the sick poor, no tickets or letters of recommendation being required to gain admission. Fifty-four thousand five hundred and fifteen patients were in attendance last year (1892), and of these 23,600 were new cases, and 11,791 accidents or emergencies. The accommodation afforded by the portion of the new hospital erected in place of the old buildings in the Caledonian Road, and which was opened in 1888 by their Royal Highnesses the Prince and Princess of Wales, having been found quite insufficient for the wants of the sick poor of the district, many urgent cases having to be turned away for want of room, the committee decided to proceed with the completion of the buildings. This work has now been accomplished at a cost of £30,000, towards which only £14,000 has been received or promised; and further contributions, therefore, are earnestly solicited to meet the calls of the contractors. Donations, either to the maintenance or building fund, will be thankfully received by the Secretary, William T. Grant.

**Guy's Hospital**, Borough, S.E.—Guy's is too old a friend of the public to need much said in its favour: only so long as it has closed wards and is crippled by poverty it is a reproach to the metropolis. The nett income of the hospital derived from its landed estates remains at £14,000 per annum less than it was before the agricultural depression, wih not present prospect of improvement. There is left, therefore, only an assured income of £25,000 to maintain the 500 beds now open, and which cannot be kept up at a less cost than £38,000 per annum. The greater part of this deficiency can only be met in the future as in the past, by liberal contributions from the public, exclusive of any further aid from the same source, required to reopen 100 beds still remaining vacant, for which there is a pressing demand. Matron, Miss Nott Bower; Superintendent, Dr. Perry.

**King's College Hospital**, Portugal Street, W.C.—That this hospital has much claim on the public for the good work it does goes without saying. Its finances are not, however, in that flourishing condition which they ought to be in, were the hospital supported as it should be. First, there is a need for money to meet the current expenses, as of assured income it has but £1,000 to meet an expenditure of £19,000. Next comes the need of subscriptions towards paying off a loan of £6,000, due to the bankers; and thirdly, a Special Five Years' Maintenance Fund of £50,000 was started in July last at a meeting held at Grosvenor House under the presidency of His Grace the Duke of Westminster, K.G. The sum at present given and promised amounts to about £6,100. The average annual receipts of the hospital during the past ten years has been about £16,500, including legacies, but the annual expenditure is over £19,000. The Five Years' Maintenance Fund is being raised to prevent the lamentable necessity of materially reducing the accommodation in the hospital. We must draw special attention to the convalescent home, which secures a period of ease for the patients sent to it from the hospital wards. As considerably over 24,000 patients are yearly admitted to the benefits of the hospital, it should surely have brought its claims for aid to the doors of many of the giving public, who, now knowing its needs, will, we feel confident, do their utmost to assist it. Secretary, Rev. N. Bromley; Sister-Matron, Miss Monk.

**Lon on Homœopathic Hospital**, Great Ormond Street, W.C.—This hospital has a well-earned reputation for efficiency and careful management. £12,000 will be required to complete the new building, now in progress at a cost of £45,000, of which the sum of £33,000 has already been contributed. The demand for the nurses trained in the hospital continues to increase. Connected with it is the Homœopathic Convalescent Home, 66, Enys Road, Eastbourne. This gives to men, women, and children who have recovered from illness the benefits of a temporary Eastbourne home, with good food and careful attention. Patients are received from all parts, as the home is not restricted to those who have been in the hospital, and adults contribute 7s. per week, children 3s. 6d. per week. Secretary, G. A. Cross; Lady Superintendent, Miss Brew.

**London Hospital**, Whitechapel Road, E.—This is the greatest hospital in this country. From this fact, and from the fact that it has been persistently attacked by a small clique, in spite of their contentions having been shown to be ill-founded over and over again, we make no doubt that the public will give largely to its funds, in testimony of their appreciation of the enormous value of the work which it does for the poor of East London. It includes special departments under eminent medical men for the treatment of all classes of disease, the number of beds devoted to children being greater than those to be met with in most children's hospitals. The character of the work done and its value to the public may be realised from the statement that the able Matron, Miss Lückes, has three assistants under her, in addition to two night superintendents, 19 day sisters, and 220 staff and proba ioner nurses. This great hospital of the East-end is in serious want of funds, as the committee depend on voluntary contributions for £40,000 a year to enable them to maintain the 640 beds which are daily occupied by urgent cases. House Governor and Secretary, Mr. G. Q. Roberts; Matron, Miss Eva C. Lückes.

**Metropolitan Hospital**, Kingsland Road, N.E.—Situated in the heart of the poor and densely populated districts of Shoreditch, Haggerston, Hackney, Bethnal Green, Hoxton, Dalston, and De Beauvoir Town, the hospital receives a very large number of accidents and casualties of all kinds, and the demands made on its resources, medical and surgical, are incessant and urgent. That the hospital is needed in its present position is shown by the fact that both the in and out patients have been steadily on the increase for several years past, the numbers treated last year being, in-patients, 894; out-patients' attendances, 71,754. In spite of this good work the committee this year were obliged to obtain £4,000 on mortgage, as the adverse balance of £3,651 on January 1st, 1892, had increased at the end of the year to £5,531. It is deplorable that the work of the charity should be crippled for lack of funds, owing to which several beds have had to be closed, leaving only 54 now available for in-patients, although there is accommodation for 160. Many urgent cases have therefore constantly to be refused admission, and unless money is immediately forthcoming it will be necessary to still further reduce the number of beds. We are sure that the public will readily respond to the committee's urgent appeal for help, and contributions should be forwarded to the Secretary, Mr. C. H. Byers, at the Hospital, or to the Bankers, Messrs. Glyn, Mills, and Co., 67, Lombard Street, E.C.

**Middlesex Hospital**, Mortimer Street, W.—This institution does a large share in the beneficent work of relief carried on so courageously by our voluntary hospitals despite all the difficulties, in their way. This is at once apparent when we say that in its 320 beds last year 2,912 patients were treated, whilst upwards of 39,000 out-patients received, free of charge, over £1,300 worth of medicines and dressings alone. The cancer wards are a distinguishing feature of the hospital, 35 beds being specially set aside for these cases, and the extra nursing, costly treatment, and unlimited dietary accorded to the sufferers from this terrible disease add largely to the expenses of the hospital. Secretary, Mr. F. Clare Melhado; Lady Superintendent, Miss Thorold.

**Miller Hospital and Royal Kent Dispensary**, Greenwich Road, S.E. Established 1783.—Opened in 1885 as a hospital in memory of the late Canon Miller, D.D.; this was the first hospital built in England with circular wards. During the year 14,322 cases were attended at the hospital, which is entirely dependent on voluntary contributions. About £800 more is required to carry on the work. Secretary, Mr. James Marks; Matron, Miss Clara Purvis.

**North-West London Hospital**, Kentish Town Road, was founded in 1878, and is the only institution of the kind in the north-west district. It has 47 beds, of which 18 are for sick children. Last year there were 555 in-patients and 39,606 attendances as out-patients. A noticeable feature in the statement of accounts is the comparatively small item for salaries of officers and other costs of management. Its admirably appointed wards are worth a visit, and the committee do well in cordially inviting visitors to see and judge for themselves. There is also a Samaritan Fund, founded by the late Mr. George Sturge, to provide nourishment and change of air for convalescents, and a soup kitchen (maintained by the Treasurer, Mr. George Herring), where about 2,000 dinners weekly are being distributed. The annual expenditure is never under £3,500, towards which the annual subscriptions yield less than £900. A large deficiency has therefore to be provided for by special benefactions. The committee very earnestly plead for assistance.

**Poplar Hospital for Accidents**, Blackwall, E.—This hospital is placed in a position in the far East-end of London, close to the Dock gates, close to great iron works, close to the largest gas works in London, and to many other large works. More accidents occur in this district than in any other, and the hospital is an absolute necessity. Yet during this last year many men and children requiring immediate admission and attention have had to be refused owing to want of room. This refusal means the carrying of a man, often with broken limbs or fractured skull, for over two miles to the nearest hospital, *i.e.*, the London, however severely injured. Added to this, there is no women's ward, and very little accommodation for children. The rebuilding of the hospital was forced on the committee by sanitary and structural defects. Of £27,000 needed for this all but £2,000 has been raised. The hospital has never been in debt, and, situated where it is, the committee think that they would not be justified in ever getting into debt. It is hoped, therefore, to raise this £2,000 before March, so that the whole hospital may be reopened, and not only a part, as at present. Last year five miles of men standing close side by side were treated for accidents only. Chairman, Hon. Sydney Holland; House Governor, Lieut.-Colonel Feneran; Matron, Miss Vacher.

**Royal Free Hospital**, Gray's Inn Road, W.C.—The Marquis of Dufferin and Ava as President of this hospital has recently issued an appeal on behalf of the committee, and calling special attention to its need at the present time for £12,000 to enable the new front building to be opened free from debt. His Excellency states that from the ruinous condition of the old front building, which was formerly a barrack for the Light Horse Volunteers, it became absolutely necessary to rebuild this portion of the hospital. The remaining portions were rebuilt some years ago, and are in excellent condition. To carry out this work, which will provide much needed isolation wards, casualty-rooms, and dispensary, and to provide a new steam laundry, an expenditure of £20,000 has been incurred, towards which sum the committee have raised £8,000, leaving a sum of £12,000 still urgently required. The old front building has already been removed. and the work of reconstruction is now in active progress, Money is therefore immediately required for the contractors. I therefore very earnestly solicit the help of those who have the means towards supplying the amount now needed, or to increase the current revenue by new annual subscriptions. The Royal Free Hospital was the first general hospital in London to open its doors freely to the sick and suffering poor without letters of recommendation from the governors. For 67 years it has adhered to this excellent system, and during that period over two millions of poor people have received within its walls the best medical and surgical advice and treatment. During the past year 26,000 persons received its benefits, either as in or out patients. The cost of maintenance was £10,551, whilst the ordinary income from all sources amounted to £5,118 only. The large deficit had to be met by utilising several considerable legacies, which had fortunately been received during that year. In the previous year, 1891, there was, however, an absolute deficiency of £3,251, which had to be met by the sale of investments. Secretary, Mr. Conrad W. Thies.

**St. George's Hospital**, Hyde Park Corner, S.W.—Occupying a commanding position, there is reason to believe that it suffers in consequence, and we hope that all who pass St. George's Hospital in future will contribute something to its funds in the course of the year. Secretary and Superintendent, Mr. C. L. Todd; Matron, Mrs. Coster.

**St. Mary's Hospital**, Paddington, has 281 beds. As these words are being written there are in its wards 281 patients. Every bed is occupied. One hundred and thirty-one men, 117 women, and 38 little children are being ministered to at this moment by its doctors and nurses. Yet during the year this great hospital has been so indifferently supported that unless £8,000 is subscribed before the close of the year the charity will be unable to pay its way. Help is wanted, and it is confidently believed that those who have the means need no other incentive to bestow them than the knowledge of such a work in such need of succour. Subscriptions will be most thankfully received by the Secretary, Mr. Thomas Ryan, at the Hospital.

**Seamen's Hospital Society (Dreadnought)**, Greenwich, S.E.—This society, the establishment of which consists of the Dreadnought Hospital at Greenwich with 235 beds, and the branch hospital in the Albert Docks with 18 beds, and two dispensaries, one in the East India Dock Road and the other at Gravesend, is treating a largely increasing number of patients. Last year there were over 16,000 sick and injured seamen under treatment in the various establishments, this being just double the number attended five years ago. Many of the cases are of exceptional interest, men having fallen from aloft while at sea and unable to obtain proper medical relief for days and often weeks, others suffering from the effects of shipwreck and exposure; while a large number have contracted diseases of the respiratory organs from the hard life that they lead. Of all men seamen are most in need of succour and assistance when ill in a strange port without friends, and often without money. Like so many other medical institutions, the Seamen's Hospital Society has suffered of late years, and the expenditure during the past twelve months has exceeded the income by about £2,000. It is gratifying to note that a great proportion of the receipts of the charity are derived from contributions from the seafaring classes who actually benefit by the treatment afforded, and the committee, therefore, very confidently appeal to the public, every individual member of which benefits by the labour and bravery of seamen, to assist this charity. Secretary, Mr. P. Mitchelli; Matron, Miss Cooke.

**University College Hospital**, Gower Street, W.—The North London or University College Hospital is mainly dependent upon voluntary contributions, the income on which it can rely being only £6,000, whilst the necessary annual expenditure is very nearly £20,000, and the present debt exceeds £7,000. The rebuilding of the hospital, too, upon a more modern plan, as soon as possible, is an urgent necessity, and for this purpose a fund was instituted in 1884, named the "Jubilee Endowment and Building" Fund, to which contributions are earnestly invited. Secretary, Mr. Newton H. Nixon.

University College Hospital.

**Westminster Hospital**, Broad Sanctuary, S.W.—Out of an expenditure of £15,000, less than £3,000 is assured to this charity, so that about £12,000 has to be made up each year in subscriptions. The expenditure for the year just about to close is nearly £5,000 in excess of the receipts, and a considerable number of the regular supporters of the hospital have either withdrawn, or reduced their usual subscriptions in consequence of "bad times." Not only is the hospital of use to those resident in the immediate neighbourhood, but as some 8,000 casualties are received within its walls, it may be inferred that others ought to take an interest in its well-being. It does an immense service to the country by training a large number of excellent nurses. Secretary, Mr. Sidney M. Quennell; Matron, Miss Pyne.

**West London Hospital**, Hammersmith Road, W. (101 beds), is the nearest for a population of nearly 500,000 persons. The accommodation for in-patients is lamentably short of the number requiring admission, and in each succeeding year the fact is brought more to the front than in its predecessor. Last year (1892) the beds were occupied by 1,713 patients, and the out-patient department was attended by 26,089 others. There is practically no endowment, and it is greatly to be feared that the excess of expenditure over income for the year now closing will be about £3,000. The board appeal for funds for the erection of a section of the contemplated extension to accommodate about 60 beds, as well as for carrying on the present work. Secretary, Mr. R. J. Gilbert; Lady Superintendent, Miss Hardy.

## Figures and Tables

**Figure f1:**
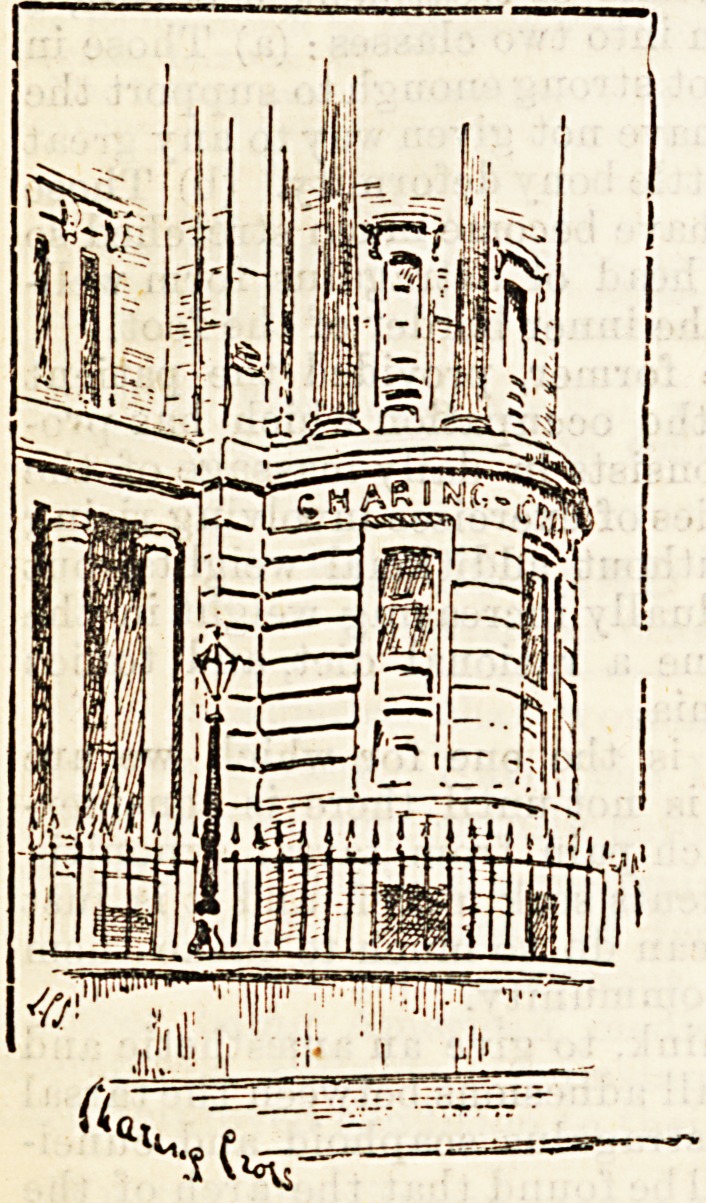


**Figure f2:**
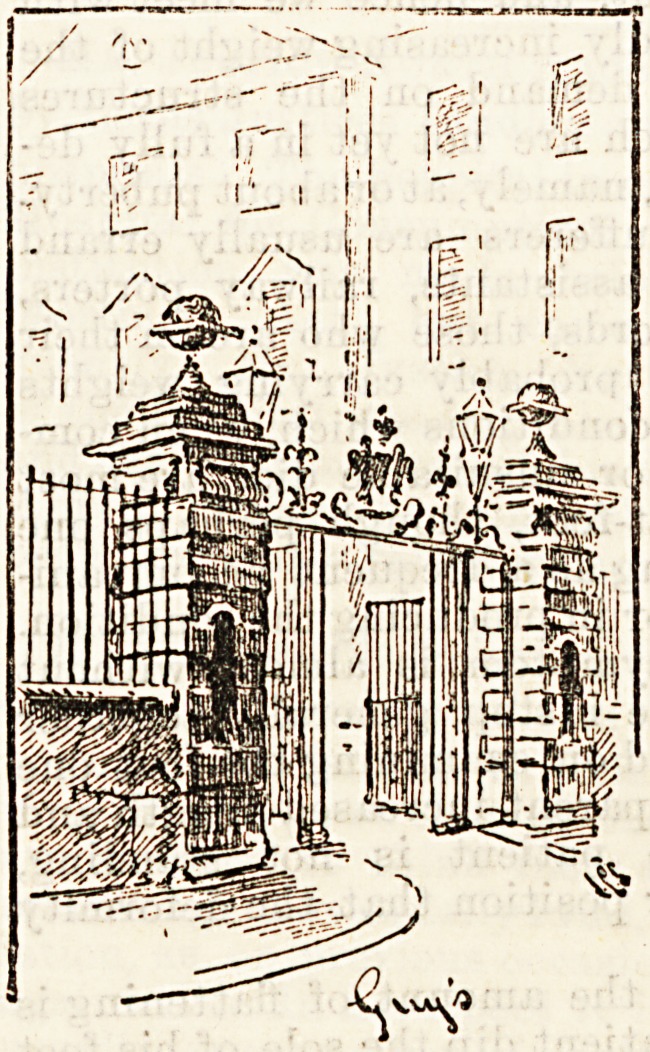


**Figure f3:**
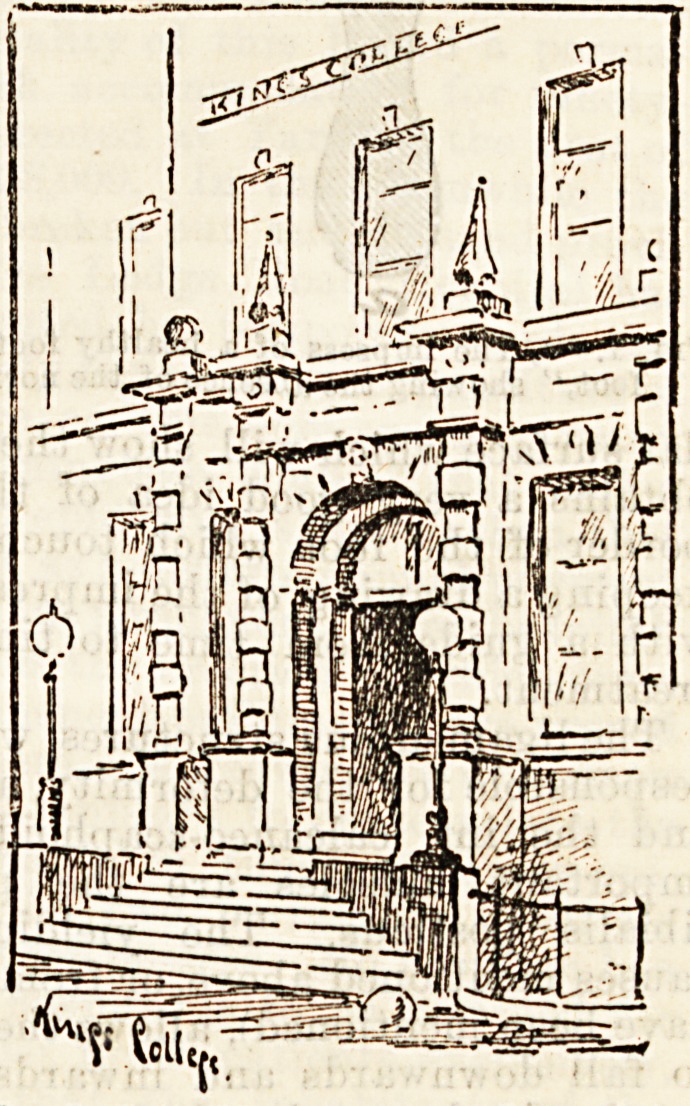


**Figure f4:**
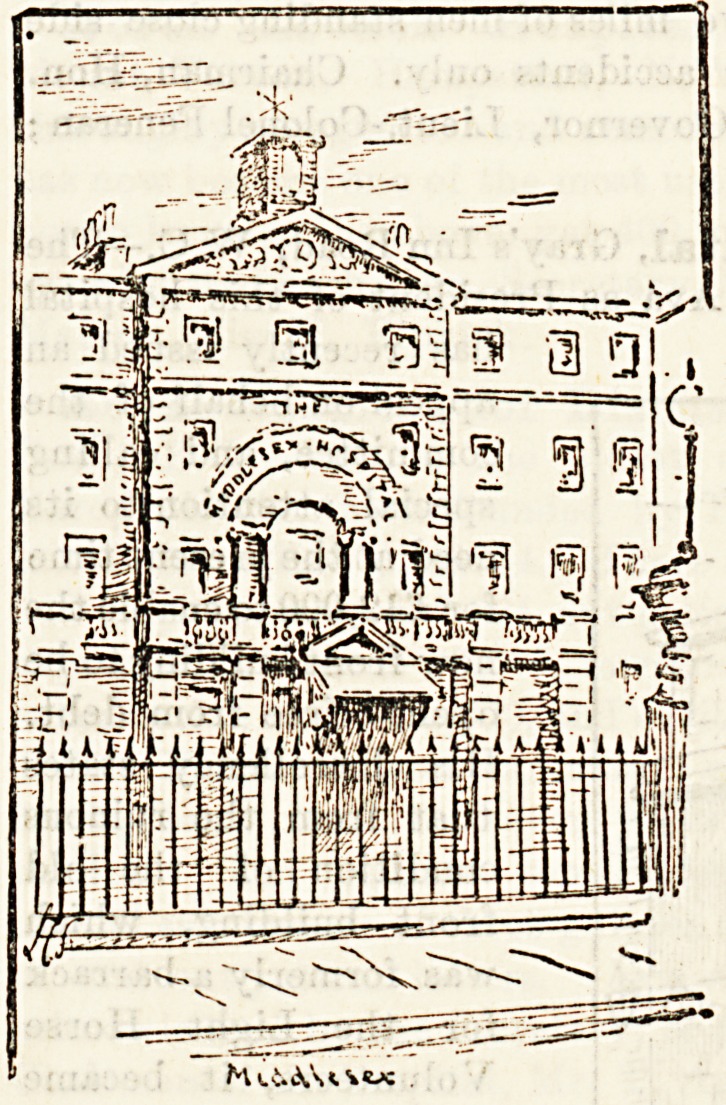


**Figure f5:**
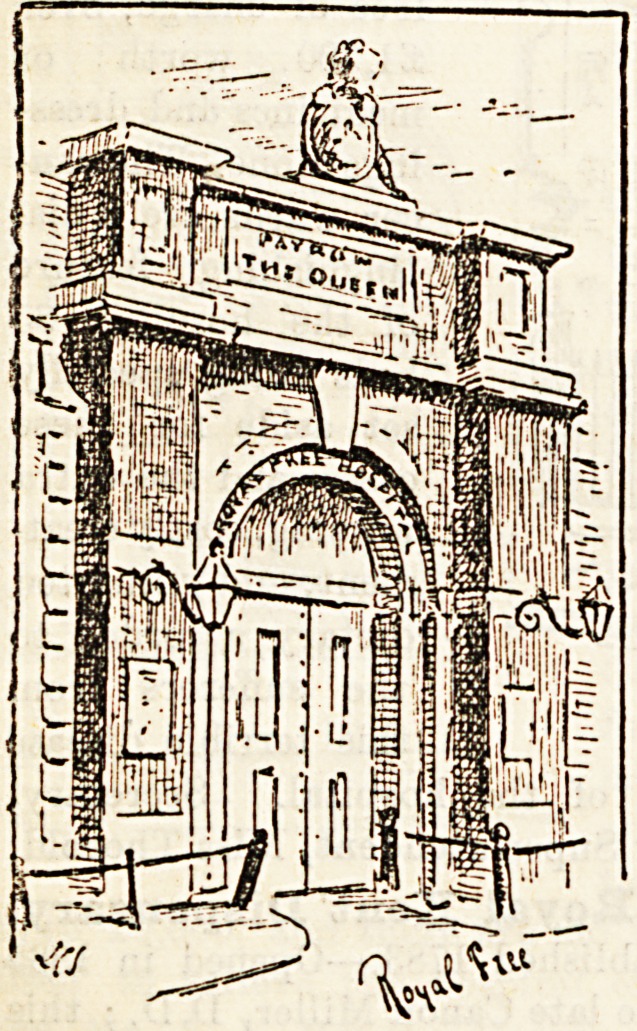


**Figure f6:**
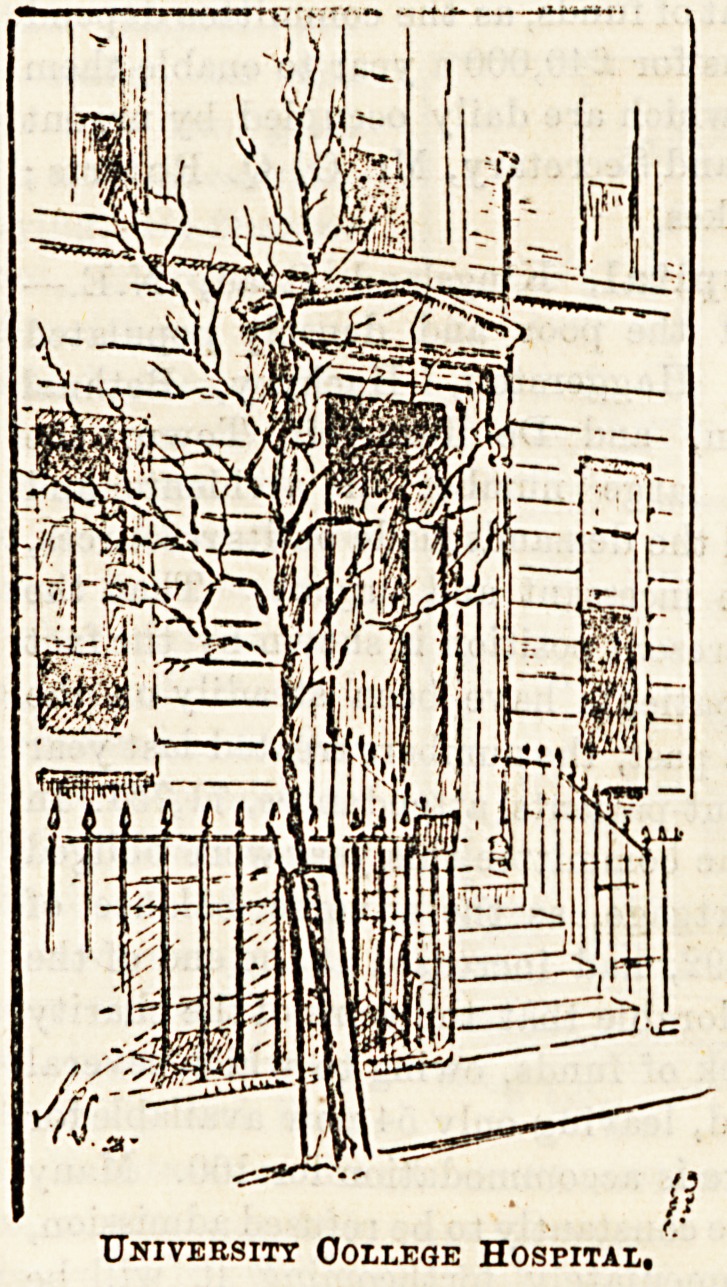


**Figure f7:**